# Regulatory B Cells in Tumor Microenvironment

**DOI:** 10.3390/cimb48010106

**Published:** 2026-01-20

**Authors:** Zhuoyan Cai, Lin Xie

**Affiliations:** 1 Institute of Organ Transplantation, Tongji Hospital, Tongji Medical College, Huazhong University of Science and Technology, Wuhan 430030, China; m15205988662_1@163.com; 2 Key Laboratory of Organ Transplantation, Ministry of Education, Wuhan 430030, China; 3 NHC Key Laboratory of Organ Transplantation, Wuhan 430030, China; 4 Key Laboratory of Organ Transplantation, Chinese Academy of Medical Sciences, Wuhan 430030, China; 5 Organ Transplantation Clinical Medical Research Center of Hubei Province, Wuhan 430030, China

**Keywords:** regulatory B cells, tumor immunity, epigenetics, metabolic remodeling, tumor immunotherapy

## Abstract

Regulatory B cells (Bregs) are integral to the tumor microenvironment (TME) and influence immune responses through the secretion of immunosuppressive cytokines such as IL-10, IL-35, and TGF-β. This review highlights recent findings on the phenotype and mechanisms of Bregs, emphasizing their dual role in regulating immune responses within the TME. Importantly, we further explored the latest advances in Breg regulatory mechanisms from the novel perspectives of epigenetics and metabolic remodeling, including the effects of DNA methylation, histone acetylation, glycolysis, and oxidative phosphorylation on Bregs. We also investigate the therapeutic targeting of Bregs, with a focus on STAT3 inhibitors such as lipoxin A4, cucurbitacins, and resveratrol, which show promising potential in mitigating the suppressive function of Bregs. Furthermore, this review provides a detailed analysis of the impact of Bregs on tumorigenesis and metastasis, emphasizing the importance of inhibiting specific immune pathways to prevent tumor escape. Finally, this review offers a prospective outlook on immunotherapy strategies based on Bregs, foreseeing a more nuanced understanding of their TME function and the evolution of targeted treatments with enhanced therapeutic efficacy.

## 1. Introduction

Tumor development is a complex process that involves not only genetic mutations within cells but also interactions with the surrounding environment. Tumor cells exhibit complex interdependence with immune cells, blood vessels, stromal cells, the extracellular matrix, and other cell types, collectively contributing to a distinct tumor microenvironment (TME). Diverse factors secreted within this milieu promote a chronic inflammatory, immunosuppressive, and pro-angiogenic environment, facilitating cancer cell adaptation and proliferation while reducing the likelihood of detection and elimination by host immunosurveillance systems [1]. As our understanding of the TME expands, the diversity of both immune and non-immune cell types identified within it increases, along with the identification of numerous biological molecules and mechanistic pathways that present potential targets for cancer therapy [2]. Simultaneously, advancements in tumor research methodologies continue to progress, with emerging evidence from studies on epigenetic and metabolic remodeling highlighting the potential for a comprehensive investigation of regulatory B cells (Bregs) [3]. In this review, we examine the role of Bregs within the TME, as reported in existing studies, investigate the genetic regulatory factors influencing Breg differentiation and function, and consider the prospective applications of Bregs in tumor immunotherapy.

## 2. Phenotypic Heterogeneity of Regulatory B Cells

The concept of regulatory B cells (Bregs) was initially introduced in 2002 by Mizoguchi et al., who identified B cells with immunosuppressive functions within an inflammatory bowel disease (IBD) model [4,5]. Over the subsequent two decades, extensive research efforts have not yet resulted in the identification of a definitive marker for the Breg lineage comparable to FoxP3 for regulatory T cells (Tregs). Although certain surface markers, such as CD19 and TIM-1, are commonly expressed on IL-10-producing B cell populations, they lack specificity for Bregs [6,7,8,9,10,11,12,13]. Bregs exhibit substantial functional heterogeneity, which is intricately linked to their tissue of origin and manifested in diverse cytokine secretion profiles. While IL-10 production is a characteristic feature of many Breg cell subsets, others are capable of secreting inhibitory cytokines such as TGF-β and IL-35, or mediating suppression through cell surface molecules such as programmed cell death ligand 1 (PD-L1). This functional diversity, in conjunction with advanced techniques such as transcriptomic analysis and multiparametric flow cytometry, has enabled the systematic characterization of Breg phenotypes across various cancers, as summarized in Table 1.

Table 1 lists Breg subsets based on their reported phenotypes, anatomical locations, effector mechanisms, and functions in different species and tumor types. Notably, there is a lack of a standardized definition and classification system for Bregs. The subsets presented are primarily categorized according to context-dependent phenotypic markers, immunosuppressive cytokine secretion, and experimentally validated functional outcomes in tumor immunity. This table underscores two key features of Breg biology: context-dependent heterogeneity, where similar phenotypes, such as human CD19^+^CD24^+^CD38^+^ B cells, may operate through distinct mechanisms in different cancers, and pronounced species-specific differences [14,15,16].For instance, CD5^+^ B10 cells are a major IL-10-producing subset in mice [17], whereas in humans, IL-10-producing Bregs are often enriched within circulating CD24^+^CD27^+^ populations [18].Beyond the subsets listed, other notable tumor-associated Breg populations have been described, including B10 cells that secrete IL-10 and suppress monocyte TNF-α production [19], immunosuppressive T2 marginal zone precursor (T2-MZP) B cells with a phenotype of CD21^+^CD23^+^IgM^+^ [20], and granzyme B (GrB)^+^ B cells capable of directly inducing T cell apoptosis in tumors [21].

**Table 1:** Different species, tumors, and Breg phenotypes and functions are involved.

Cancer Type	Species	Breg Phenotype	Effector Function	Location	Function	Reference
Thymic carcinoma	Human	CD19^+^CD25^+^	IL-10	Cancer tissue	Induce the generation of Treg	[22,23]
HNSCC						
BC	Mouse	PD-1^−^PD-L^+^CD19^+^	PD-1^−^PD-L^+^CD19^+^	Spleen and PB	Inhibit T cell proliferation and IFN-γ production	[24]
	Human	CD24^hi^CD27^+^	IL-6, TNF-α	M-LN	Enhance BC resistance	[25]
		CD19^+^CD81^hi^CD25^+^	TGF-β	PB	Induce Treg and evade tumor immunity	[26]
GC	Human	CD19^+^CD24^hi^CD38^hi^	IL-10	Cancer tissue	Promote GC immune escape	[16]
		CD19^+^CD24^hi^CD27^+^CD27^+^CD10^−^	TGF-β1			[18]
Melanoma	Mouse	B220^+^CD23^+^IgM^hi^CD21^hi^	N/A	TDLN	Aggregates in TDLN and promotes tumor growth	[27]
	Human	CD19^+^CD24^hi^CD38^−^ lgM^+^	TGF-β1, PD-L1	PB	Induce the generation of Treg	[28]
CRC	Mouse	CD19^+^lgA^+^	PD-L1, IL-10, TGF-β	Cancer tissue	Inhibition of CD8^+^ T cell dif-ferentiation and activation	[29]
	Human	CD19^+^B220^−^CD20^−^IgD^−^CD38^+^CD27^+^CD138^−^	TGF-β1	Tumors and PB	Induce the generation of Treg	[3]
HCC	Rat	CD19^+^IL-10^+^	IL-10	PB	Promote the development of HCC	[30]
	Human	CD19^+^CD5^+^ IL-10^+^		Cancer tissue	Promote HCC development	[31]
Others in human	PDAC	CD38^+^CD19^+^	IL-10, IL-35	Cancer tissue	Inhibition of NK cells, CD8^+^ T cells	[19]
	TSCC	CD19^+^IL-10^+^	IL-10		Induce the generation of Treg	[32]
	IBCa	CD19^+^CD24^+^CD38^+^	PD-L1		Induce Treg and evade tumor immunity	[33]
	Glioma	TGF-β^+^	TGF-β		Suppression of CD8^+^ T cell activity	[34]

HNSCC: head and neck squamous cell carcinoma; HCC: hepatocellular carcinoma; PDAC: pancreatic ductal adenocarcinoma; BC: breast cancer; M-LN: metastatic lymph node; GC: gastric cancer; TSCC: tongue squamous cell carcinoma; IBCa: invasive breast carcinoma; PD-L1: programmed cell death ligand 1; PB: peripheral blood; CRC: colorectal cancer; TDLN: tumor-draining lymph node.

In conclusion, the phenotypic and functional diversity of Bregs highlights their complex and multifaceted roles in tumor immunoregulation. The lack of specific surface markers and a consensus classification system represents a significant limitation. Overcoming this challenge through improved definitions and standardized categorization is crucial for advancing fundamental understanding and for developing targeted therapeutic strategies tailored to specific tumor immune microenvironments.

## 3. Determinants of Regulatory B Cell Differentiation

Bregs are not innately present in the body; they differentiate in response to different incentives in the immune microenvironment at different stages of the B cell differentiation process. The specific differentiation process of Bregs has not yet been fully elucidated. This process integrates signals from B cell-intrinsic receptors, soluble factors in the tumor microenvironment (TME), and a network of downstream transcriptional regulators [35]. These signals are transduced through a complex network of transcriptional, epigenetic, and metabolic programs that ultimately define the regulatory identity and function of the cell.

### 3.1. Molecular Signals Driving Breg Differentiation

Breg differentiation is initiated by a confluence of signals from B cell-intrinsic receptors and the surrounding cytokine milieu, which are integrated by downstream transcription factors.

B cell surface receptors provide critical activation and differentiation signals to B cells. The engagement of Toll-like receptors (TLRs), such as TLR9, sensing microbial patterns directly promotes IL-10 production [36]. In the tumor microenvironment (TME), signaling through the B cell receptor (BCR) drives the expression of the immunosuppressive cytokine IL-35 in pancreatic cancer [37]. Concurrently, co-stimulatory signals, notably the interaction between CD40 on B cells and CD40L on T cells, can induce transcriptional reprogramming that enhances the regulatory capacity [38]. Extrinsic soluble factors provide critical cues. For example, lipopolysaccharide (LPS) stimulates TLR4 and induces the secretion of other factors, such as RANTES, TNF-α, and TGF-β, which indirectly foster a Breg-supportive niche [39]. Furthermore, cytokines such as B cell-activating factor (BAFF) exhibit dose-dependent effects; lower concentrations preferentially promote the expansion of IL-10-producing Breg cell subsets [40].

Downstream transcription factors translate these external signals into stable regulatory programs. For instance, the aryl hydrocarbon receptor (AhR) directly binds to the *Il10* promoter. This binding activates transcription while simultaneously suppressing proinflammatory gene programs, thereby defining the IL-10-producing Breg identity [41]. Furthermore, in the hypoxic TME, hypoxia-inducible factor 1α (HIF-1α) is critical for IL-10 production, partly by driving glycolytic metabolic reprogramming [42]. Intriguingly, HIF-1α also represses the expression of BACH2, a key transcription factor for B cell development [43]. This interaction between HIF-1α and BACH2 suggests a metabolic-transcriptional coupling layer that may influence the development or function of immunosuppressive B cells in the TME.

### 3.2. Epigenetic Regulation of Breg Identity

Epigenetic modification is a way of finely regulating gene expression that has a profound effect on the differentiation, proliferation and function of Bregs without altering the DNA sequence [44]. These modifications include methylation and demethylation of DNA, various chemical modifications of histones, and the regulatory effects of non-coding RNAs. These mechanisms act synergistically to determine the active state of genes, which in turn play important roles in a series of key biological processes, such as cell growth, apoptosis, differentiation, aging, and immune response [45]. While the classification of B cell subsets provides a foundational understanding of their functional diversity, the epigenetic modifications of Bregs at the gene level further determine their role in tumor immunity.

**Figure 1:**
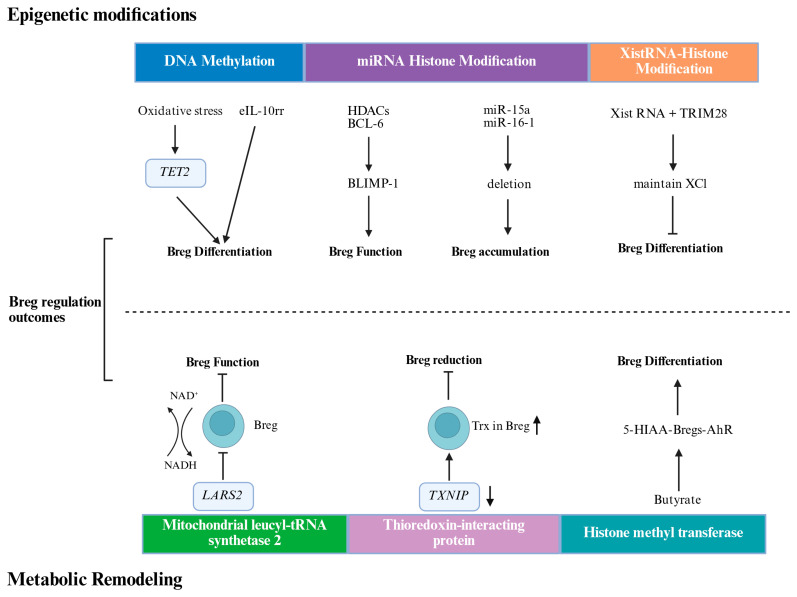
Epigenetic modifications and metabolic remodeling regulate Breg function and differentiation. eIL10rr, the early IL-10 regulatory region; TET2, Ten-eleven translocation-2; Bcl-6, B cell lymphoma 6; HDACs, histone deacetylases; TRIM28, Tripartite Motif Containing 28; XCI, X-chromosome inactivation; NAD, Nicotinamide Adenine Dinucleotide; LARS2, leucyl-tRNA synthetase 2; Trx, Thioredoxin Reductase; TXNIP, Thioredoxin-interacting protein. Created in BioRender. Ye, D. (2026) https://BioRender.com/roykymt.

DNA methylation is a well-recognized, stable, and heritable epigenetic modification that plays a key role in the regulation of gene expression (Figure 1). A key discovery identified a demethylated cis-regulatory element 4.5 kb upstream of the Il10 transcription start site, termed the early IL-10 regulatory region (eIL10rr) [46]. The methylation status of this region distinguishes B cells capable of rapid IL-10 production from those requiring sustained stimulation. The demethylase enzyme ten-eleven translocation-2 (TET2) is activated by oxidative stress in the HCC microenvironment. This activation promotes IL-10 production, which subsequently drives tumor progression. Consequently, the adoptive transfer of TET2-deficient Bregs inhibited HCC progression, highlighting TET2 as a potential therapeutic target [47].

In addition to DNA methylation, histone modifications add further layers of regulatory control. B cell activation induces histone-modifying enzymes, such as activation-induced cytidine deaminase (AID), and histone modifications, such as H3K4me3, H3K9ac, and H3K14ac, are induced during B cell activation and are crucial for immune functions, such as somatic hypermutation (SHM)and class switch DNA recombination (CSR)in Bregs. Studies have shown that B lymphocyte-induced maturation protein 1 (Blimp-1) also regulates the differentiation of Bregs, with its expression being regulated by B cell lymphoma 6 (Bcl-6) and histone deacetylases (HDACs) [48].

ncRNAs, including long noncoding RNAs (lncRNAs) and microRNAs (miRNAs), play a significant role in B cell differentiation and antibody immune response by regulating gene expression [49]. Researchers have primarily focused on miRNAs for their role in non-coding RNAs in B cell differentiation and antibody immune response. miRNAs are single-stranded RNA molecules, 21 to 25 nucleotides long, that significantly impact cellular functions by finely regulating post-transcriptional and post-translational gene expression [50,51,52]. Studies have confirmed that Deletion of MIR15A and MIR16-1 promotes the accumulation of specific B cells, which produce immunosuppressive effects on CD8^+^ T cells through the secretion of IL-10 and TGF-β [29].

X-chromosome inactivation-specific transcript (Xist RNA) is a key long-stranded non-coding RNA that plays an important role in the regulation of X-chromosome inactivation (XCI). Xist RNA deposits epigenetic modifications by recruiting a variety of protein complexes to the X-chromosome, reshaping X-chromosome structure, and silencing gene transcription in specific nuclear regions, which is essential for early embryonic development of XCI. In different subpopulations of B cells, Xist RNAs exhibit specific expression and localization patterns that have important implications for Breg function and identity [53].

Using Gene Set Enrichment Analysis (GSEA), Howard Y. Chang et al. [54] found that in atypical B cells (IgD^+^CD27^+^CD11c^+^atypical B cells, ABCs)from patients with systemic lupus erythematosus (SLE), Xist RNA expression levels were higher than in healthy donors. This Xist-dependent regulation of gene expression involves histone deacetylation and is associated with specific protein complexes such as TRIM28 (Tripartite Motif Containing 28), which, as a key cofactor of Xist RNA, participates in the maintenance mechanism of XCI by affecting the proximal suspension of the promoter of RNA Pol II and deacetylation of H3K27, and thus participates in the immune regulation of B cells.

### 3.3. Metabolic Reprogramming in Breg Function

Cellular metabolism is not merely a housekeeping function but is a critical regulator of immune cell fate. In Bregs, metabolic reprogramming is intrinsically linked to differentiation and suppressive capacity [55]. For instance, in patients with colorectal cancer, a specific Breg subset was found to express high levels of mitochondrial leucyl-tRNA synthetase 2 (LARS2), which promoted NAD^+^ regeneration and enhanced TGF-β1 production, correlating with accelerated tumor growth [3].Similarly, Breg cell differentiation depends on mitochondrial electron transport chain activity and moderate levels of reactive oxygen species (ROS). This process is controlled by the thioredoxin (Trx) and thioredoxin-interacting protein (TXNIP) redox system, which is dysregulated in B cells from patients with SLE [56].Finally, short-chain fatty acids (SCFAs), such as butyrate, produced by the gut microbiota, can modulate Breg differentiation and function, demonstrating a link between diet, the microbiome, and Breg-mediated immunity [57].

In summary, Breg differentiation is a multi-layered process orchestrated by the TME. It begins with the integration of external molecular signals, which are interpreted by a core network of transcription factors. This signaling cascade initiates profound and stabilizing changes at both the epigenetic level, which locks in the regulatory phenotype, and the metabolic level, which fuels the suppressive machinery. A critical area for future research is understanding the interplay between these regulatory layers. Elucidating these integrated pathways is essential for developing therapies that can precisely disrupt Breg differentiation while sparing beneficial B cell functions.

## 4. Interaction of Breg with the Tumor Microenvironment

The TME is characterized by chronic inflammation, in which a complex network of inflammatory mediators orchestrates the recruitment and function of diverse immune cell populations. Within this intricate milieu, Bregs emerge as pivotal players. This chapter dissects the bidirectional relationship between Bregs and the TME, focusing first on how Bregs modulate the tumor landscape and, second, on how the TME shapes Breg differentiation and function [58].

### 4.1. Influence of Breg on TME

Bregs play a multifaceted role in the tumor microenvironment with complex and diverse functions. Bregs in the TME promote tumor growth by secreting immunosuppressive factors, such as IL-10, TGF-β, and IL-35, which inhibit the activity of T cells and create an immunosuppressive microenvironment [59].

#### 4.1.1. Effect of Bregs on Tumor Cells in TME

Bregs employ a multifaceted strategy to foster a pro-tumorigenic environment, primarily through the secretion of immunosuppressive cytokines and direct cell–cell interactions. The secretion of IL-10 is a hallmark of Breg-mediated suppression. For instance, in human HCC, a PD-1^hi^ Breg subset was shown to curtail tumor-specific T-cell immunity via IL-10 production, thereby promoting tumor progression [60]. This pro-tumorigenic role is not confined to HCC; in ovarian cancer, elevated frequencies of IL-10-secreting B cells in ascites correlate with disease advancement, decreased IFN-γ^+^CD8^+^ T-cell response, and increased presence of FoxP3^+^ Tregs [61,62].These clinical observations were substantiated by preclinical models. In a murine 4T1 breast cancer model, tumor-induced Bregs (tBregs) were found to secrete IL-10 and TGF-β, which drives the conversion of conventional CD4^+^ T cells into immunosuppressive FoxP3^+^ Tregs [24].

In addition to soluble factors, direct intercellular contact is another key mechanism. In HCC, Bregs have been demonstrated to directly promote tumor cell proliferation and invasion through the CD40-CD154 signaling pathway, a finding validated in both in vivo and in vitro settings [63]. Furthermore, specific Breg populations, such as PD-1/PD-L1^+^CD19^+^ Bregs in patients with breast cancer, exert potent T-cell inhibition, highlighting the clinical relevance of these direct interactions [24]. The discovery of unique Breg-associated gene signatures in bladder cancer (BLCA) further underscores their potential as biomarkers for prognosis and immunotherapy response predic In a genetically engineered mouse model of pancreatic cancer (KRas^G12D^-PDEC), CD19^+^CD1d^hi^CD5^+^ Bregs were demonstrated to facilitate tumor growth and expansion through the production of IL-35, reinforcing the diverse cytokine-dependent pathways Bregs can exploit [64].

#### 4.1.2. Effect of Bregs on Immune Cells in TME

The interaction between B cells and other immune constituents of the TME is remarkably complex, revealing a dichotomous role for B cells in tumor immunity. While this review focuses on the immunosuppressive functions of Bregs, it is crucial to acknowledge that certain B cell subsets can actively promote antitumor responses.

For example, a subset of ICOSL^+^ B cells bolsters anti-tumor immunity by increasing the effector T cell to Treg ratio [65]. Similarly, studies led by Joshi et al. demonstrated that tumor-antigen-specific germinal center (GC) B cells are instrumental in driving the proliferation and differentiation of follicular helper T cells (T_FH_). These T_FH_ cells secrete IL-21, which enhances granzyme B production in tumor-infiltrating CD8^+^ T cells, thereby potentiating cytotoxic antitumor activity [66]. This study elegantly elucidated a key molecular axis (B cell-T_FH_-IL-21-CD8^+^ T cell) for B cell-mediated tumor rejection.

In stark contrast, Breg cell subsets exert potent inhibitory effects. A compelling example is found in PDAC models, where activation of the Stimulator of Interferon Genes (STING) pathway within B cells unexpectedly triggers their regulatory functions. This STING-IL-35 axis in Bregs leads to suppressed NK cell proliferation and a diminished antitumor response, ultimately promoting tumor growth [67]. This highlights how the same signaling pathway (STING), often associated with antitumor immunity, can be co-opted by Bregs to achieve an opposite, pro-tumorigenic outcome.

### 4.2. Regulatory Effects of TME on Bregs

The TME actively induces the differentiation of Bregs to create a self-perpetuating immunosuppressive loop. Tumor cells can directly induce Breg differentiation via various mechanisms. For instance, glioma cells release Placental Growth Factor (PlGF) to induce a Breg phenotype [34]. Similarly, HCC-derived exosomes drive the conversion of B cells into suppressive TIM-1^+^Bregs [68]. A recent study using a murine colorectal cancer model further elucidated this mechanism. Tumor-derived exosomes (MC38-Exos) directly induced an immunosuppressive Breg phenotype by upregulating IL-10 and TGF-β. Mechanistically, MC38-Exos suppress B cell receptor (BCR) signaling pathways and activate the NF-κB pathway, ultimately inhibiting B cell proliferation and promoting their suppressive functions [69]. Finally, metabolites within the TME, such as those from the arachidonate 5-lipoxygenase (ALOX5) pathway, can drive B cell differentiation into Bregs [70].

The TME and Bregs engage in complex, bidirectional crosstalk that overwhelmingly favors tumor progression [71,72]. Bregs, induced by TME-derived signals, deploy a range of suppressive mechanisms to dampen antitumor immunity. A critical future challenge is to precisely delineate the signals that dictate the balance between the regulatory and effector B cell fates. This knowledge could unlock novel therapeutic strategies to selectively foster antitumor B cell responses.

## 5. Targeting Regulatory B Cells for Cancer Immunotherapy

The growing understanding of Breg-mediated immunosuppression has opened new therapeutic avenues aimed at dismantling this barrier to effective antitumor immunity, as shown in Table 2 [73].

### 5.1. Inhibiting Breg Differentiation and Function

One strategy is to prevent B cells from acquiring a regulatory phenotype or blocking their suppressive functions. Peroxisome proliferator-activated receptor delta (PPARδ), which is highly expressed in IL-10-producing Bregs, acts as a key regulator of their development. Consequently, PPARδ inhibitors can effectively suppress IL-10^+^ Breg development and function, providing a targeted antitumor approach [79]. Another potential vulnerability lies in metabolic programming. Blockade of the electron transport chain (ETC) with agents such as rotenone or antimycin A has been shown to inhibit IL-10 expression in B cells following TLR or CD40 stimulation, which are critical signals for Breg differentiation.

### 5.2. Depleting Regulatory B Cell Populations

An alternative approach involves the selective reduction in the number of Bregs. For instance, the MEK inhibitor cobimetinib was found to reduce Breg populations in tumor-draining lymph nodes (TDLNs) while preserving anti-tumor humoral immunity in a colorectal cancer model [27]. Mechanistically, this may involve the disruption of chronic BCR signaling, which is required for Breg maintenance. Other chemical modulators can also recalibrate the balance between regulatory and effector B cells. The TLR9 agonist CpG ODN significantly reduced CD20^low^ Bregs while activating antitumor B cells, leading to inhibited lung metastasis in a 4T1 mouse model [80].

### 5.3. Targeting Key Signaling Pathways in Bregs

Beyond depleting the cells themselves, a more nuanced strategy involves targeting the intracellular signaling cascades that empower Bregs. The STAT3 pathway is a central node for Breg-mediated immunosuppression, as its activation is essential for IL-10 and TGF-β production [76]. Several compounds, including lipoxin A4 and resveratrol, inhibit STAT3 activation in Bregs, thereby abrogating their suppressive functions [81,82,83]. Targeting intercellular communication is another powerful approach. This was powerfully illustrated in a recent study on glioblastoma (GBM), an immunologically ‘cold’ tumor type. Spatially resolved transcriptomics identified the TGFβ pathway, driven primarily by myeloid cells, as the key signal promoting a regulatory state in tumor-infiltrating B cells. Crucially, pharmacological or genetic blockade of TGFβ signaling synergized with PD-1 inhibition to eradicate tumors in a B-cell-dependent manner. This dual therapy rescued CD8^+^ T-cell cytotoxicity from Breg-mediated suppression and promoted plasmablast differentiation. This study not only underscores the critical role of TGFβ in maintaining the Breg niche but also provides a strong rationale for combining TGFβ blockade with checkpoint inhibitors to overcome immunotherapy resistance [78]. Similarly, combining a PD-1 inhibitor with an IL-18 inhibitor (IL-18BP) in a pancreatic cancer model effectively blocked the Breg-CTL suppressive axis and inhibited tumor growth [77].

In conclusion, targeting Bregs represents a burgeoning frontier in cancer immunotherapy. Current preclinical strategies are focused on three primary fronts: inhibiting Breg development and function via PPARδ or ETC inhibitors, selectively depleting Breg populations with MEK inhibitors or CpG ODNs, and disrupting core signaling pathways, such as STAT3 or CD40. As detailed in Table 1, most of these approaches remain in the preclinical stages of investigation. The path to clinical translation will require a deeper understanding of target specificity, potential off-target effects on beneficial B cell functions, and the development of robust biomarkers to identify patients who are most likely to benefit from Breg-targeted therapies. The combination of Breg-depleting agents with established immunotherapies, such as ICB, is a particularly promising avenue for future clinical trials.

## 6. Summary and Outlook

As described above, Bregs play a dual role in the tumor microenvironment in inhibiting or promoting immune responses by secreting cytokines such as IL-10, IL-35, and TGF-β. Decades of research have gradually elucidated the immunomodulatory function of Bregs in multiple tumor models. Although the exact phenotype and specific markers of Bregs are not fully defined and the mechanisms of their interaction with the tumor microenvironment (TME) are still being explored, research on the role of Bregs in tumor development is ongoing. Recently, an increasing number of studies have shown that epigenetic modification and metabolic remodeling are associated with the promotion of tumor development by Bregs. The mechanisms of epigenetic modification and metabolic remodeling of Bregs and their roles in antitumor immune responses are not fully understood. Future studies should further investigate the role of Breg cells in tumor progression from the perspective of epigenetic and metabolic remodeling, characterize the phenotype and function of Breg cells in different tumor microenvironments, and elucidate the mechanisms leading to Breg cell production in patients and the role of Breg cells in evading immune surveillance.

## Figures and Tables

**Table 2:** Therapeutic modulators targeting Bregs.

Modulator	Target/Mechanism of Action	Level of Experimental Validation	Known Off-Target Effects and Safety Considerations	References
**PPARδ Inhibitors**	Inhibits PPARδ, a key transcription factor for Breg development and IL-10 production.	Murine cancer models, human B cells (in vitro)	Systemic metabolic disturbance and impaired cutaneous wound healing, given the pleiotropic role of PPARδ in lipid metabolism and tissue repair.	[70]
**ETC Inhibitors**	Block oxidative phosphorylation, inhibiting IL-10 production in B cells stimulated via TLR7 or CD40L.	B cell culture (In vitro)	Broad mitochondrial toxicity in all aerobic tissues, leading to unacceptable systemic organ toxicity and limiting therapeutic utility.	[38]
**MEK Inhibitors**	Reduces Breg numbers, possibly by disrupting chronic BCR signaling.	Mouse CRC (in vivo)	MEK regulates cell proliferation; inhibitors commonly cause rash, diarrhea, and visual disturbances.	[74]
**CpG ODN**	Activates TLR9, selectively depleting CD20^low^ Bregs while activating anti-tumor B cells.	Mouse BC (in vivo)	Potent systemic immune activation risks dose-limiting cytokine release syndromes, such as fever and hypotension.	[36]
**Resveratrol**	Non-specific inhibitor of multiple pathways including STAT3, NF-κB, PI3K/Akt.	Mouse melanoma model (in vivo)	Despite its pleiotropic anti-inflammatory profile, extremely low oral bioavailability represents a paramount translational challenge.	[75]
**STAT3 Pathway Inhibitors**	Specifically inhibit STAT3 phosphorylation in Bregs, reducing secretion of immunosuppressive cytokines like IL-10 and TGF-β.	In vitro studies and mouse models	STAT3 is a master regulator for most immune cells and epithelial repair; inhibition carries a high risk of global immune dysfunction and compromised tissue regeneration.	[76]
**CD40/CD40L Blockade**	Blocks CD40-CD154 interaction between Bregs and tumor cells.	Mouse HCC (in vivo)	Blockade of the CD40 pathway weakens antitumor immunity while simultaneously promoting tumor progression.	[63]
**IL-18BP+** **α** **PD-1/PD-L1**	Neutralizes IL-18 to relieve CTL suppression, synergizing with PD-1/PD-L1 blockade to enhance T cell function.	Mouse PDAC (in vivo)	Combination immunotherapy effectively inhibits the tumor growth and metastasis but increases the risk of immune-related pneumonitis, and colitis.	[77]
**TGFβ Signaling Inhibition**	Rescued CD8^+^ T-cell cytotoxicity from Breg-mediated suppression and promoted plasmablast differentiation.	Human GBM (in vitro).	Inhibition of TGF-β signaling is associated with cardiotoxicity (valvulopathy, arteriopathy), anemia, impaired wound healing, and bone abnormalities in nonclinical studies.	[78]

## Data Availability

No new data were created or analyzed in this study. Data sharing is not applicable to this article.
